# Key person ethical decision-making and substandard drugs rejection intentions

**DOI:** 10.1371/journal.pone.0229412

**Published:** 2020-03-19

**Authors:** Xiaohong Ren, Xiaoyan Wang, He Sun

**Affiliations:** 1 School of Pharmaceutical Science and Technology, Tianjin University, Tianjin, China; 2 School of Chemistry and Chemical Engineering, Tianjin University of Technology, Tianjin, China; Shandong University of Science and Technology, CHINA

## Abstract

Substandard drugs are a major public health issue worldwide. Key person such as the Qualified Person in China and Europe is responsible for rejecting substandard drugs during the manufacturing stage. This study applies the Hunt-Vitell ethical decision-making model to study their rejection intentions on substandard drugs. Using the experimental vignette methodology, two scenarios were developed to represent different levels of deviation from regulations in pharmaceutical manufacturing. Responses from 204 Chinese key persons show a decline in deontology, ethical judgment, and rejection intention, and an increase in teleology in the minor deviation scenario, in comparison with the major deviation scenario. The results from the two scenarios show that the Hunt-Vitell ethical decision-making model is well fitted to explain substandard drug rejection intentions. Organizational and occupational commitments have a significant positive impact on deontological evaluation. Whereas, occupational commitments have a significant negative impact on teleological evaluation. This study suggests that strengthening occupational commitment can significantly affect key person’s rejection intentions of substandard drugs.

## Introduction

Medicines can treat or prevent illnesses, but substandard drugs could harm or even kill patients seeking aid. The World Health Organization (WHO) defines a substandard medicine as an out-of-specification product authorized by national regulatory authorities yet fails to meet national and/or international quality standards or specifications. Substandard drugs can result in serious consequences, such as failing to prevent or cure a disease and causing needless suffering for the patient. Substandard antimicrobials contribute to antimicrobial resistance through the development of drug-resistant mutations and pathogen transmission[1].

**Fig 1:**
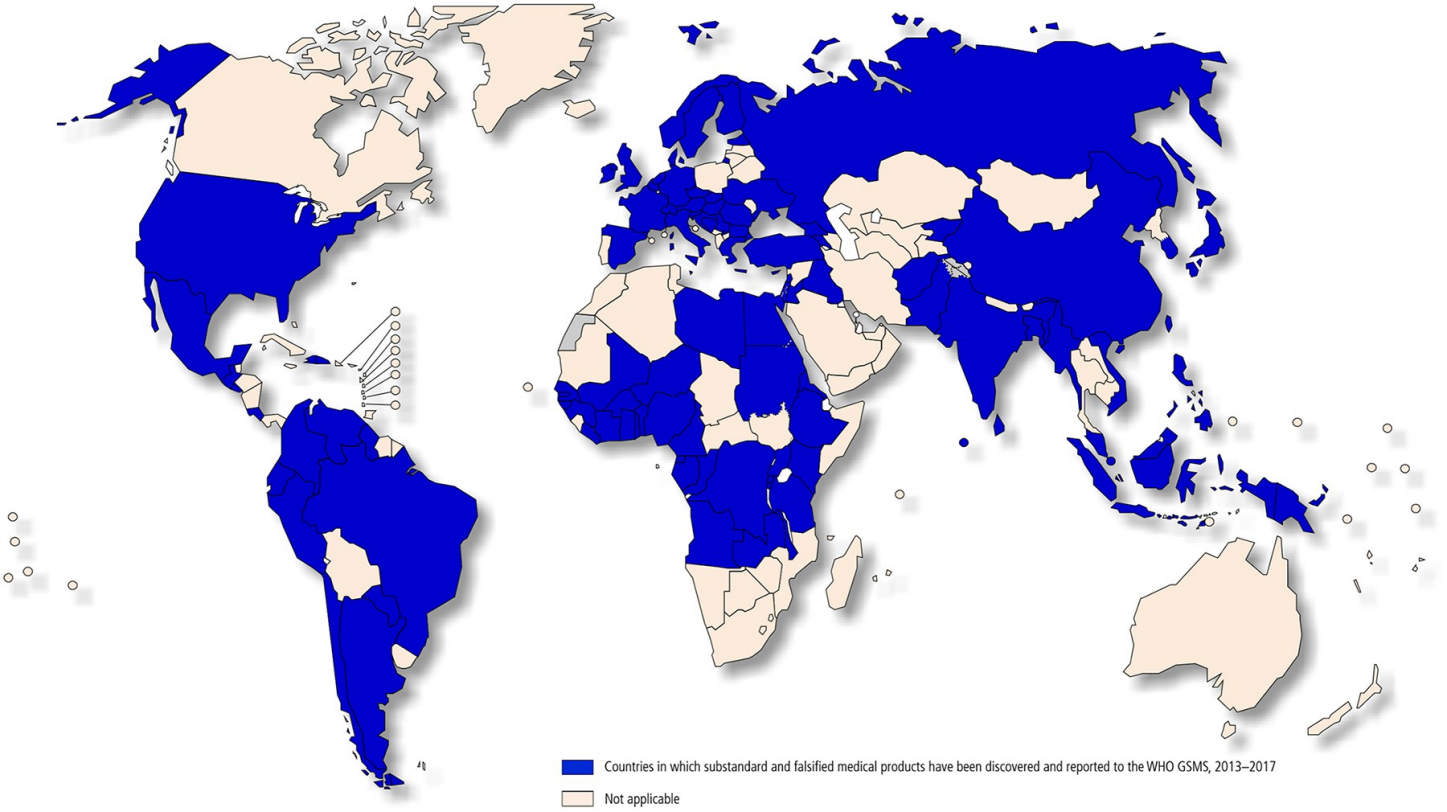
Countries in which substandard and falsified medicinal products have been reported to WHO, 2013–2017[1].

Unfortunately, substandard drugs have become a major public health issue worldwide[2–4]. According to WHO’s Global Surveillance and Monitoring System (GSMS), substandard and falsified medicinal products have been discovered in many countries (Fig 1).

China is also a hard-hitting area for substandard drugs. Uncovered in July 2018, the production of freeze-dried human rabies vaccine in Changchun Changsheng Biotechnology Co., Ltd. (Changsheng) violated regulations with major deviation in its manufacturing processes from NDA approved protocols. Changsheng retained expired stock in the finished production, changed product batch number, modified production date, and falsified manufacturing records. The social impact of this major vaccine event is one of the most significant in China to date.

### Substandard drugs

Drug quality depends on many factors, including manufacturing bias, re-marking of expired drugs, and degradation during storage[5]. In China, the Chinese Pharmacopoeia provides specifications for a wide spectrum of drugs, specifically indicating that the compliance of manufacturing processes to the requirements of Good Manufacturing Practice (GMP) is the basis for quality drugs. In short, the term “quality drug” carries two meanings: the drug’s testing results meet specifications, and its manufacturing process complies with GMPs.

A type of substandard drug, those that meet test result specifications but created with manufacturing processes that deviate from GMPs, harms patients and are difficult to detect. Drug testing is based on statistical sampling and does not represent every tablet or capsule. Drugs that lack strict manufacturing controls meet testing specifications because poor-quality pills are not sampled by chance and are therefore not revealed.

Substandard drugs produced by a poor manufacturing processes are very likely discovered through on-site inspection. After the Changsheng incident, the China National Medical Products Administration (NMPA) started to conduct flight inspections of manufacturers. According to the 2018 NMPA Annual Inspection Report, a total of 234 drug manufacturing on-site inspections were performed. However, considering the 7,000 registered pharmaceutical manufacturers in China, the number of inspections amount to less than 4%. To fundamentally solve the problem of substandard drugs, in addition to external oversight, internal controls for drug manufacturing are of paramount importance, and the person responsible for internal controls is the critical determinant.

**Fig 2:**
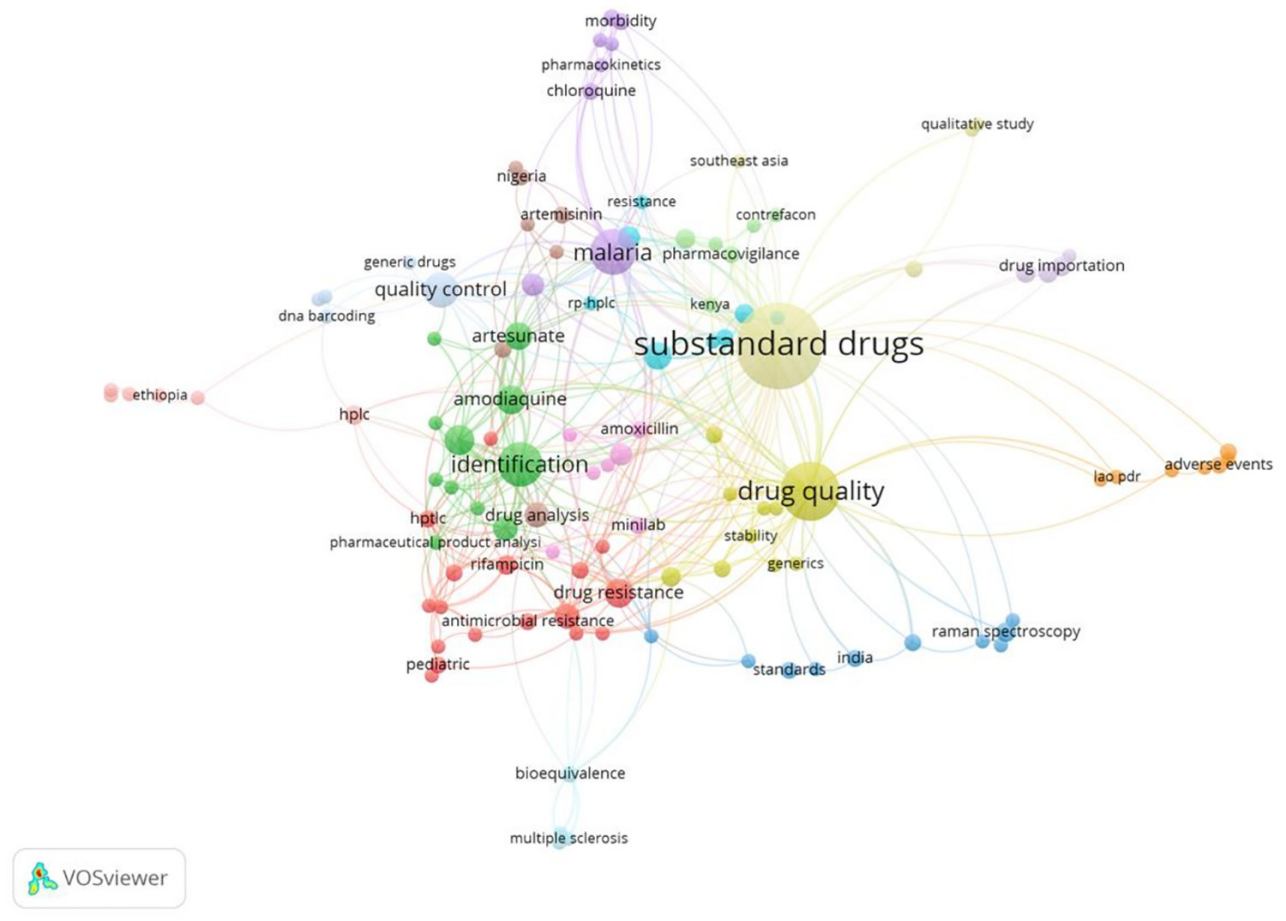
Map of keywords of groups on substandard drugs during 1985–2019.

In recent years, researchers use bibliometric methods to analyze studies for recognizing research status and trends. The keywords occurrence network of VOSvoewer reflects study hotspots and research trends of a certain domain[6]. A total of 557 papers were identified by collecting the studies on substandard drugs from the Web of Science Core Collection database with key words including substandard drug/s, medicine/s, and medicinal product/s. Author Keyword were used to analyze the study area of these articles. Fig 2 shows the most prominent application areas between 1985 and 2019. Each color in the figure represents one cluster of author keywords. The intensity of a color indicates occurrence weights; the more intense the color, the more times the keyword appeared in the lists of keywords. The keywords show three prominent clusters, analysis technical, resistance, and regulation. These clusters show focus of substandard drug studies on technical and regulation.

### Key person in quality control during the drug manufacturing stage

When quality control systems are mandatory, which China introduced in 2011, there is a key person, called qualified person (QP), overseeing this system and is responsible for drug quality control throughout the manufacturing stage.

According to China’s current GMP article 2, “No batch of products can be released without the approval of the QP.” Stipulated in GMP article 25, the QP carries two main responsibilities. First, the QP is a full-time employee of the manufacturer and participates in quality management activities, such as establishment of the quality system, self-inspection, and external quality audits. Second, the QP is responsible for ensuring that “the production and testing of each batch of released products are in accordance with related regulations and registration specifications” during drug release.

Therefore, the key person should thoroughly understand the drug manufacturing process and clearly grasp the “hidden” defects of a drug. The key person’s decision is the last quality control before a drug enters the market. Ideally, they would reject substandard drugs according to relevant laws and regulations.

This study is interested in the key person’s consideration process when they release substandard drugs, the elements that affect the key person’s release decision, and the prevention of releasing substandard drugs by the key persons.

### Key persons releasing substandard drugs as an Ethical Issue

With professional education and experience, the key person is highly aware of the risks that substandard drugs pose to patients. Thus, releasing good quality drugs is a professional and ethical obligation. When the key person is faced with substandard drugs, they make a judgment and take one of two possible actions: to release or to reject the drug. When the key person chooses to release substandard drugs, they also choose to infringe on the interests of patients. Lefkowitz states that ethics is “the study of how one should properly live one’s life, especially with respect to behavior toward others”[7]. Therefore, a key person releasing substandard drugs should be considered as an ethical issue.

Research on substandard drugs is from either the technical or regulatory perspective [8][9][10], which is considered as external management. Thus far, research has yet to view the substandard drugs issue from the key persons ethical perspective, which is an internal perspective. Compared with the external management, self-quality control by manufactures is another effective way to solve the substandard drug problem, where every task is performed by individuals, highlighting the fundamental importance of their possible issues. Compared with the technical and regulatory study, the ethical perspective focuses on the individual’s issues. Therefore, the study of ethical decision making in key persons is an attempt to solve substandard drug problems from a fundamental standpoint.

## Theoretical background and hypotheses

### Ethical decision theories and models

An ethical decision is defined as a decision that is both legal and morally acceptable. Conversely, an unethical decision is either illegal or morally unacceptable[11]. The ethical decision theory has been extensively studied in many fields, such as business, pharmacy, and accounting[12–18].

In 1986, Rest proposed a four-stage ethical decision theory comprising ethical awareness, ethical judgment, intention, and behavior. This model suggests that, as a starting point, individuals first recognize the existence of ethical issues in the workplace[11]. Next, the individual will enter the judgment step, which is related to the ethicality of the situation. Then, individuals form an ethical intention, which will primarily determine their action[19]. Many other factors, such as individual characteristics, moral philosophy, way of thinking, environmental factors, and organization factors, may be included in these four stages, thus directly or indirectly affecting individual ethical decision-making (EDM)[11, 20]. There are several EDM models: contingency model, person-situation interactionist model, issue-contingent model, and the Hunt and Vitell (H-V) model. The contingency model focuses on external factors[21]. The person–situation interactionist model pays attention to the interaction between individual and situational factors[22]. The issue-contingent model links special challenges present in organizational settings to ethical agents[11]. Among ethics models, Hunt and Vitell’s model (H-V) explains the individual decision-making process in detail by positing that the decision maker evaluates personal behavior and its consequences[23]. One purpose of this research is to discover how the key person’s decision is made without considering external factors such as organization; therefore, the H-V model is best fit for it.

### The Hunt and Vitell model

In 1986, Hunt and Vitell developed an ethical model designed for marketing and other aspects of business that covered the 3-stages of Rest’s theory. The H-V model increases the scope and flexibility of business ethics by linking EDM to more than a single moral perspective[24]. The H-V model has undergone strict empirical tests and has shown that it explains and predicts EDM[18, 25–27]. This model is now frequently employed by ethics researchers as a general theory[28, 29].

Hunt and Vitell believed that moral decision-making is rooted in the social environment and individual experience. When the individual perceives an ethical dilemma, they make a decision based on long-term experiences that have affected previous ethical judgments. The H-V model enlists the concepts of “deontology” and “teleology” in ethical philosophy[30]^.^ Deontological evaluation involves comparing an action against “predetermined deontological norms representing personal values or rules of behavior”[31] ascertaining the moral correctness of the behavior’s characteristics rather than the value it brings. In contrast, teleological evaluation covers the magnitude, probability, and desirability of the consequences of an action, together with the importance of “each stakeholder group” [30, 32] placing value on the moral of the behavior’s consequences[28]. Individuals strictly follow their moral criteria, or moral code, completely ignoring the consequences of their actions[33, 34]. Whereas, others may focus on the consequences and neglect ethical criteria[35]. In most cases, the comprehensive assessment of both “deontology” and “teleology” affect judgment[23, 36]. Hunt and Vitell suggest that ethical intentions and behavior are directly influenced by ethical judgments[28, 30].

### Commitment theories

Organizational commitment is considered as a psychological state regarding an employee’s relationship, attachment, and identity with their organization and occupation[37], [38]. The greater the individual organizational commitment, the more their identity depends on their organization[39]. There are three established forms of organizational commitment: affective, continuance, and normative[39, 40], respectively representing the “want”, “need”, and “ought” towards continuing to working for their organization[37]. Compared to continuance and normative commitments, affective commitment should be more positively related to organizational citizenship[40].

Occupational commitment refers to having a positive attitude toward one’s occupation, reflecting a strong sense of identity with, and participation in the profession[41]. Meyer verified the difference between occupational and organizational commitments, in that they contribute independently to the prediction of professional activity and work behavior. In addition, he shows that occupational commitment, similar to organizational commitment, has affective, continuance, and normative forms.

We focus on the affective dimension of these two types of commitments for the following reasons. First, studies show that having a similar occupation, such as clinical supervision, is relevant for predicting affective commitment, but not for continuance or normative commitments[42]. Second, affective commitment is associated with organizationally relevant outcomes, such as turnover, job performance, and employee well-being[43], suggesting its association with teleological evaluations. In this study, we employ affective organizational commitment and affective occupational commitment to measure key person’s dual commitment.

### Study hypotheses

According to the H-V model, the person performs deontological and teleological evaluations after he/she identifies that a situation contains ethical content. In the deontological evaluation, the person assesses the inherent rightness or wrongness of the behavior form his/her values and beliefs of right and wrong[36]. In the pharmaceutical industry, there are very clear regulation on the rightness and wrongness of a manufacturing actions. A deontological key person would act based on his/her belief and decide whether the substandard drugs should be released. In the teleological evaluation, the person assesses rightness or wrongness of the behavior from the consequences that may result from each possible alternative[30]. The teleological evaluation process focuses on four constructs: the perceived consequences of each alternative for various stakeholder groups, the probability that each consequence will occur to each stakeholder group, the desirability or undesirability of each consequence, and the importance of each stakeholder group[28]. Therefore, a key person with teleology orientation would decide to reject or release substandard drugs after weighing all possibilities and consequences. Higher deontology leads to stricter ethical evaluation[31], whereas higher teleology focuses on the results of an ethical decision[36].

Based on this, we propose the following hypotheses:

H1: Key person’s deontological evaluations will have a significant positive effect on ethical judgments toward substandard drug rejections.
H2: Key person’s teleological evaluations will have a significant negative effect on ethical judgments toward substandard drug rejections.

The H-V theory proposes that both ethical judgments and intentions should better predict behaviors when the ethical issues are central, rather than peripheral[26]. The theory holds that ethical judgments sometimes differ from intentions because teleological evaluations also directly affect intentions. That is, teleological evaluation would prioritize consequences over ethicalness. On this basis, we expect that:

H3: Key person’s ethical judgment has a significant positive effect on the intentions toward substandard drug rejections.
H4: Key person’s teleological evaluation has a significant negative effect on the intentions toward substandard drug rejections.

Both organizational and occupational commitments are interdependent and connected[44]. An individual can value both the organization and their occupation[45], but scenarios that challenge the occupation’s moral standards may cause organizational commitment to clash with occupational commitment[46]. This study focuses on key persons, a special group with dual identities. As full-time employees, they have organizational commitment, however, as professionals registered with the government, they are responsible for releasing quality medicine and have occupational commitment.

Organizations aiming for short-term interest may request the release of substandard drugs, placing an ethical dilemma on the key person. In this circumstance, the level of organizational commitment has a significant effect on deontological and teleological evaluations. With this information, we propose:

H5: The organizational commitment of a key person has a significant positive effect on deontological evaluation.
H6: The organizational commitment of a key person has significant negative effect on teleological evaluation.

Jeffery found that Taiwanese accountants with a high level occupational commitment are inclined to follow occupational rules when faced with conflicts between the profession and an organization while ignoring the gains and losses for involved parties[47]. Similarly, the key person with high occupational commitment should be inclined to comply with drug regulations. Therefore, we propose:

H7: The occupational commitment of a key person has a significant positive effect on deontological evaluations.
H8: The occupational commitment of a key person has a significant negative effect on teleological evaluations.

**Fig 3:**
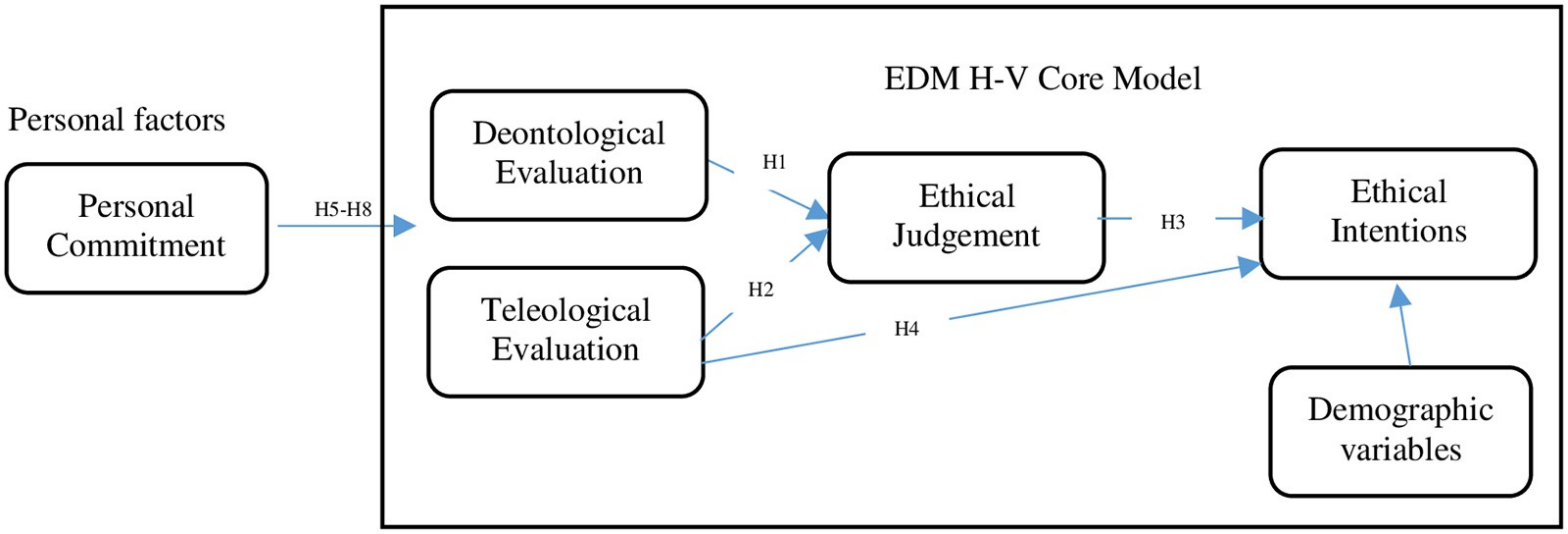
Theoretical framework.

According to Craft’s 2013 literature review, the most commonly used demographic factors for individual ethical decisions include gender (38 articles), age (14 articles), education and work experience (27 articles)[48]. Therefore, this study selects these variables as personal factors. The theoretical model is shown in Fig 3.

## Experimental vignette methodology and vignette build

The experimental vignette methodology (EVM) presents participants with carefully constructed, realistic scenarios to assess dependent variables, including intentions, attitudes, and behaviors[49, 50]. The combination of the vignette technique with traditional survey can reveal a respondents’ beliefs, attitudes, and judgments[49]. Using vignettes allow investigators to add additional background information and details into ethical issues, basing the responses in ethical research on high quality data[50, 51]. The “paper people study” is an important type of EVM, presenting participants with vignettes, typically in the written form, then asking them to make explicit decisions, judgments, choices, or express behavioral preferences[50]. This type of EVM has been widely used in EDMs[52, 53], which is employed for this study.

### Scenario design

Some validity vignettes had been used for EDM in some areas[54, 55], but no vignette is available for EDM on substandard drug release in neither English nor Chinese literature. Two vignettes were constructed based on inspection findings from U.S. Food and Drug Administration (FDA) and China NMPA and an extensive literature. These vignettes included substandard drugs where the manufacturing process deviated from GMPs while its testing results met specifications. We believe that if a key person rejects this type of substandard drugs, they should also reject other types of substandard drugs.

After interviewing drug manufacturing experts, two factors were selected to construct the vignettes: the degree of manufacturing process deviation and stakeholder importance. The degree of deviation from regulations is a continuous variable divided into two levels, major and minor. The importance of stakeholders is a dichotomous variable that focuses on the organization and the patients, resulting in two scenarios (two degrees of deviation × one importance of stakeholder).

Scenario 1 is where the key person protects organizational interests in the presence of major deviation. We define a major deviation as when key mixing process deviates from registered manufacturing process and the batch manufacturing record is fabricated. This situation seriously deviates from regulation, the products present a high risk for patients, and it is likely to be detected in subsequent tests.

Scenario 2 is where the key person protects organizational interests in the presence of minor deviation. The manufacturer used equipment intended to replace the same type of existing equipment, but the new equipment was used for commercial production without qualification. This is considered as a minor deviation from GMPs.

These scenarios were originally written in Chinese. They were translated and reviewed by a senior pharmaceutical expert. The scenarios are described in Appendix.

### Scenario content validity

When such scenarios are incorporated into the design of a research study, their content validity (CV) is assessed before application[56]. CV describes the extent to which the components of an instrument represent and are relevant to meeting its objective[57]. The quantitative evaluation of CV is performed through expert assessment and a series of indicators are calculated[58]. Six experts on drug manufacturing and five management experts assessed the final scenario drafts to test our CV[59]. The sentences of the scenarios were split into seven items for measurement (represented with ①-⑦) and were evaluated with four aspects: “description is clear,” “description is credible,” “manipulation variable is obvious,” “description is consistent with the purpose”[59].

Experts that agreed to participate in the evaluation received a document containing a cover letter (study aims, method, and description of how to assess content validity) and a content validity assessment form. The items were scored using a four-point scale (1 = strongly disagree, 4 = strongly agree). The CV indicator and criteria are listed in Table 1, the assessment results are listed in Table 2. All results meet the criteria.

**Table 1:** CV indicator and criteria.

	Indicator	Calculation	Criteria
1	IR (interrater agreement)	The sum of the number of entries with an expert rating of 1 or 2 and the number of entries with an expert rating of 3 or 4 divided by the total number of entries.	Not less than 0.7 [60].
2	I-CVI (item-level CVI)	For each entry, give the number of experts with a score of 3 or 4 divided by the total number of experts participating.	Not less than 0.78 (more than six experts) [60].
3	Modified kappa K*	K* = (I-CVI-Pc)/(1-Pc)	0.40~0.59 ok; 0.60~0.74 good, more than 0.74 excellent [61].
4	S-CVI/UA (scale-level CVI)	The number of entries with an expert rating of 3 or 4 divided by the total number of entries.	Not less than 0.8 [60].

**Table 2:** CV results of scenario 1 & 2.

**Scenario 1**	**Items**	**Number of Experts rating 3–4**	**I-CVI**	**Pc**	K*	**Evaluation result**
Description is clear^a^ Description is credible^a^	1	6	1.00	0.016	1.00	Excellent
	2	6	1.00	0.016	1.00	Excellent
	3	6	1.00	0.016	1.00	Excellent
	4	6	1.00	0.016	1.00	Excellent
	5	6	1.00	0.016	1.00	Excellent
	6	6	1.00	0.016	1.00	Excellent
	7	6	1.00	0.016	1.00	Excellent
Manipulation variable is obvious^b^	3	5	1.00	0.041	1.00	Excellent
	5	5	1.00	0.041	1.00	Excellent
	6	5	1.00	0.041	1.00	Excellent
Description is consistent with the purpose^c^	1	10	0.92	0.005	0.92	Excellent
	2	11	1.00	0.000	1.00	Excellent
	3	11	1.00	0.000	1.00	Excellent
	4	11	1.00	0.000	1.00	Excellent
	5	11	1.00	0.000	1.00	Excellent
	6	11	1.00	0.000	1.00	Excellent
	7	11	1.00	0.000	1.00	Excellent
**Scenario 2**	**Items**	**Number of Experts rating 3–4**	**I-CVI**	**Pc**	K*	**Evaluation result**
Description is clear^a^ Description is credible^a^	1	6	1.00	0.016	1.00	Excellent
	2	6	1.00	0.016	1.00	Excellent
	3	6	1.00	0.016	1.00	Excellent
	4	6	1.00	0.016	1.00	Excellent
	5	6	1.00	0.016	1.00	Excellent
	6	6	1.00	0.016	1.00	Excellent
	7	6	1.00	0.016	1.00	Excellent
Manipulation variable is obvious^b^	3	5	1.00	0.041	1.00	Excellent
	5	5	1.00	0.041	1.00	Excellent
	6	5	1.00	0.041	1.00	Excellent
Description is consistent with the purpose^c^	1	10	0.92	0.005	0.92	Excellent
	2	11	1.00	0.000	1.00	Excellent
	3	11	1.00	0.000	1.00	Excellent
	4	11	1.00	0.000	1.00	Excellent
	5	11	1.00	0.000	1.00	Excellent
	6	11	1.00	0.000	1.00	Excellent
	7	11	1.00	0.000	1.00	Excellent

Note: Number of experts participating in the evaluation: a = 6, b = 5, c = 11.

## Methods and measure

Expert interviews were conducted during the initial questionnaire design stage. Experts included NMPA inspectors responsible for on-site inspections, management professors, the heads of pharmaceutical companies, QPs, quality department staff, production department staff, and R&D staff at pharmaceutical manufacturing sites. In the questionnaire, the title of position was changed to QP to fit Chinese regulations.

Organizational and occupational affective commitment were measured with the scale established by Meyer (1993), which included 12 items. The original Cronbach’s alpha is 0.82[37]. The 12-item scale by Reidenbach (1990) was used to measure the degree of deontology and teleology. The original average Cronbach’s alpha is 0.80[54]. The four-item scale by Hartikainen (2004) was used to measure ethical judgment. The original Cronbach’s alpha ranged from 0.78 to 0.92 for different scenarios[62]. A three-item scale by May & Pauli (2002) was used to measure intention. The original Cronbach’s alpha ranged from 0.88 to 0.92 for different scenarios[63]. These scales are commonly used in social research and EDM research.

The questionnaire is a self-rated survey in an electronic version along with a cover letter stating the detailed information of the research goal and overview (not including any specific research hypotheses) was provided with the questionnaire to participants with emphasis on voluntary and anonymous participation. In the cover letter, researchers seriously guaranteed and committed to academic morality, such as information confidentiality of the survey and the statement that all data were solely used for this research. Participants were required to answer questions truthfully. If they did not accept the survey, they can withdraw from the survey at any time. Demographic characteristics, including age, gender, education, and service time (years) for their current company. In addition to basic personal information in the questionnaire, participants reported the extent to which they agree on the statement on a 1–7 Likert scale (1 = Strongly disagree, 7 = Strongly agree) in two scenarios.

An online recruitment was implemented by two methods between June and July of 2019. One is from several pharmaceutical professional communities in which QPs joined. If interested, they could voluntarily and anonymously join the survey. A multiple-choice question (“Are you a qualified person?”) was firstly presented in this questionnaire to identify whether the participants is a QP or not. The second method is by inviting QPs online from the China Qualified Persons Association communication. The two data collection methods were jointly performed to ensure a large coverage of sophisticated and knowledgeable professionals in this area. In both ways, the researcher first introduced the research goal and overview and the voluntary and anonymous online participation. Statements of the cover letter were repeated for clarity. Except for the first question of whether the participant is a QP, other parts of the questionnaire are identical. A small gift was sent to participants after they completed the survey with a valid response. A total of 227 QPs, from the two methods, participated in the study and 204 of them responded with valid data, demonstrating a final response rate of 89.87%.

This survey received responses from QPs from 25 of 34 provinces in China, covering a majority of provinces. 54.9% of the participants were male, 43.6% were 30–40 years old, 58.8% had a bachelor’s degree, 42.2% QPs were employed in their current company for more than 10 years. The distribution of the final samples is uniform. Detailed demographics are listed in Table 3.

**Table 3:** Descriptive statistics of QP.

		Number	Percentage
Gender			
	Male	112	54.9%
	Female	92	45.1%
Age			
	20–30	23	11.3%
	30–40	89	43.6%
	40–50	66	32.4%
	Over 50	26	12.7%
Education			
	College	59	28.9%
	Bachelor	120	58.8%
	Master	24	11.8%
	Doctor	1	0.5%
Employment length			
	Less than 3 years	48	23.5%
	3–5 years	28	13.7%
	5–10 years	42	20.6%
	More than 10 years	86	42.2%

## Results

The validity test shows Kaiser-Meyer-Olkin (KMO) = 0.858 and Sig = 0.000, which demonstrates good questionnaire validity. Cronbach’s alpha = 0.779 demonstrates good reliability.

### Pearson correlations

Correlations among the variables in scenario 1 and 2 are shown in Table 4. The results show that organizational commitment (COR), occupational commitment (COC), deontological evaluation, ethical judgment, and reject intention are significantly positively correlated, and teleological evaluation is significantly negatively correlated with reject intention.

**Table 4:** Pearson correlations of scenarios (n = 204).

Scenario 1	Organizational commitment	Occupational commitment	1-Deontological evaluation	1-Teleological evaluation	1-Ethical judgment	1-Reject intention
Organizational commitment	1					
Occupational commitment	.403**	1				
1Deontological evaluation	.306**	.392**	1			
1Teleological evaluation	−.177*	−.308**	−.607**	1		
1Ethical judgment	.232**	.389**	.619**	−.658**	1	
1Reject intention	.355**	.346**	.602**	−.650**	.642**	1
Scenario 2	Organizational commitment	Occupational commitment	2-Deontological evaluation	2-Teleological evaluation	2-Ethical judgment	2-Reject intention
Organizational commitment	1					
Occupational commitment	.403**	1				
2Deontological evaluation	.267**	.362**	1			
2Teleological evaluation	−.184**	−.352**	−.673**	1		
2Ethical judgment	.237**	.373**	.808**	−.751**	1	
2Reject intention	.254**	.317**	.754**	−.731**	.782**	1

*Correlation is significant at the 0.05 level (two-tailed);

**Correlation is significant at the 0.01 level (two-tailed).

### Structural equation modeling (SEM) analysis of Scenario 1

SEM analysis is employed to test and improve the measurement models for personal commitment, ethical philosophies, ethical judgment, and intention. SEM was used to test the hypotheses, maximum-likelihood estimation was employed to analyze the covariance matrices, and our model also considered covariance paths connecting variables that may share common variance sources[64]. Statistical analyses for this study were performed with SPSS 18.0 and AMOS 22.0. Seven indices were used to assess the model’s fitness to the data: absolute fit measures indices are goodness-of-fit index (GFI) >0.90 and root mean square error of approximation (RMSEA) <0.06; incremental fit measures indices are incremental fit index (IFI) >0.90, comparative fit index (CFI) >0.90, Non-Normed Fit Index (NNFI) >0.90; parsimonious fit measures indices are Normed chi-square (NC) between 1 and 3, parsimony goodness-of-fit index (PGFI) >0.50 [65].

#### Model estimation

Results for the final structural model are: GFI = 0.903, RMSEA = 0.053, IFI = 0.946, CFI = 0.945, NNFI = 0.931, NC = 1.575, and PGFI = 0.661. These values are above the limits; therefore, we conclude that the model explains the data.

#### Hypothesis testing—Main variables

**Fig 4:**
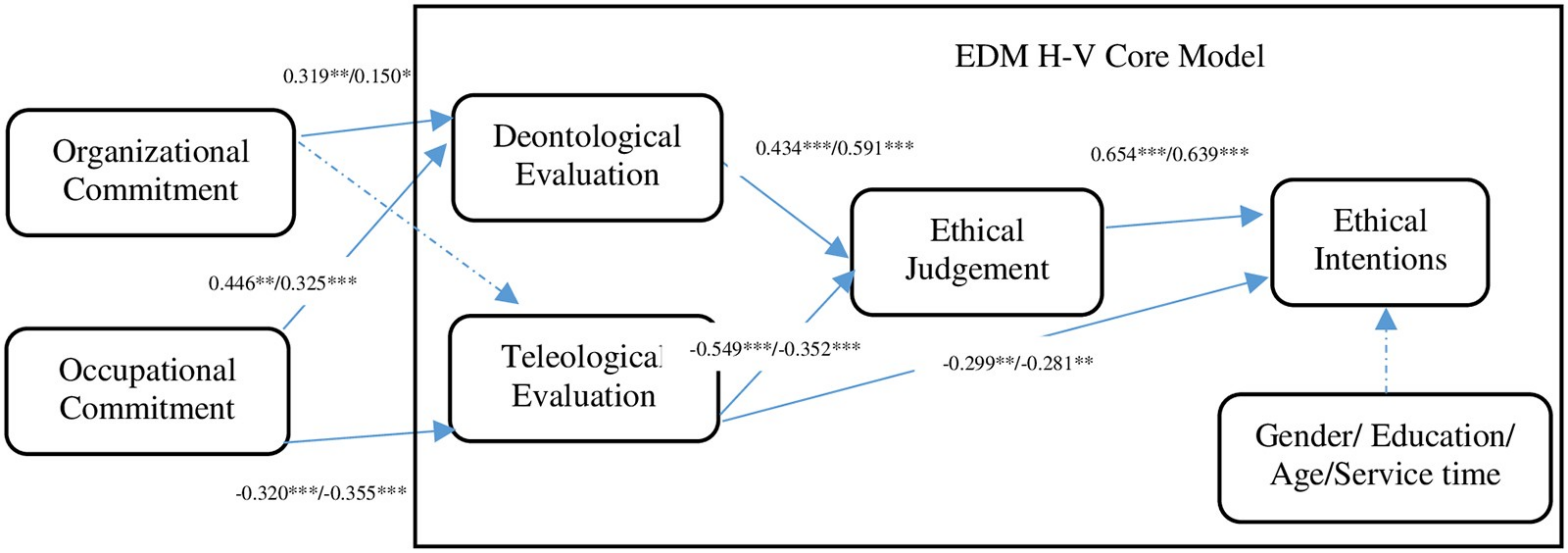
Final influencing factors and paths in scenario 1 and 2. Notes: The former numbers are results for scenario 1 and the latter numbers are for scenario 2.

The SEM results are listed in Table 5. The reject intention was positively influenced by ethical judgment and negatively influenced by teleological evaluations. Ethical judgment was affected by a combination of deontological and teleological evaluations. Deontological evaluations were positively influenced by both occupational and organizational commitments, with the former having a larger effect. The teleological evaluations were negatively affected by occupational commitment. The influencing factors and paths for substandard drug released under deviations from regulations are shown in Fig 4. Demographic variables (age, gender, education, and employment length) have no significant effect on reject intention.

**Table 5:** Hypothesis testing—Main variables of Scenario 1.

Hypothesis				S.E.	C.R.	P	Standard Estimate	Result
H1	Deontological evaluation	→	Ethical judgment	.388	3.881	***	.434	Accepted
H2	Teleological evaluation	→	Ethical judgment	.056	-8.235	***	−.549	Accepted
H3	Ethical judgment	→	Reject intention	.119	5.936	***	.654	Accepted
H4	Teleological evaluation	→	Reject intention	.084	-3.262	**	−.299	Accepted
H5	Organizational commitment	→	Deontological evaluation	.041	2.595	**	.319	Accepted
H6	Organizational commitment	→	Teleological evaluation	.112	-.474	.635	−.039	Rejected
H7	Occupational commitment	→	Deontological evaluation	.042	3.023	**	.446	Accepted
H8	Occupational commitment	→	Teleological evaluation	.094	-3.953	***	−.320	Accepted

Note:

*P < 0.1,

**p < 0.01,

***p < 0.001.

### SEM analysis of Scenario 2

#### Model estimation

The final structural model produced: GFI = 0.902, RMSEA = 0.055, IFI = 0.959, CFI = 0.958, NNFI = 0.946, NC = 1.607, and PGFI = 0.642. These values were above the limits, we conclude the model explains the data.

#### Hypothesis testing—Main variables

The SEM results are shown in Table 6. The reject intention was influenced by ethical judgment and teleological evaluations. Ethical judgment was affected by deontological evaluation and teleological evaluations. Deontological evaluation was positively influenced by both occupational and organizational commitments, with the former having a larger effect. Occupational commitment negatively impacted teleological evaluations. The influencing factors and paths for substandard drug release under deviations from regulations are shown in Fig 4. Demographic variables (age, gender, education, and employment length) have no significant effect on reject intention.

**Table 6:** Hypothesis testing—Main variables of Scenario 2.

Hypothesis				S.E.	C.R.	P	Standard Estimate	Result
H1	Deontological evaluation	→	Ethical judgment	.190	5.607	***	.591	Accepted
H2	Teleological evaluation	→	Ethical judgment	.088	-3.599	***	−.352	Accepted
H3	Ethical judgment	→	Reject intention	.118	5.689	***	.639	Accepted
H4	Teleological evaluation	→	Reject intention	.100	-2.656	**	-.281	Accepted
H5	Organizational commitment	→	Deontological evaluation	.086	1.662	*	.150	Accepted
H6	Organizational commitment	→	Teleological evaluation	.149	-.620	.535	-.048	Rejected
H7	Occupational commitment	→	Deontological evaluation	.072	3.539	***	.325	Accepted
H8	Occupational commitment	→	Teleological evaluation	.120	-4.661	***	-.355	Accepted

Note:

*P < 0.1,

**p < 0.01,

***p < 0.001.

## Discussion

### EDM H-V core model

The H-V core model was verified in both scenarios. Deontological evaluation in the H-V moral decision model, teleological evaluation, and ethical judgment have a significant impact on reject intentions. These results are consistent with the H-V model. Therefore, we conclude that the H-V core model for EDM is suitable to explain the key person’s intention at rejecting substandard drugs.

This model explains that Chinese QP approach their decision from both deontology and teleology perspectives when faced with substandard drugs. If the key person has a high degree of deontological evaluation, they will make strict ethical judgments and is more likely to reject substandard drugs. With a higher degree of teleological evaluation, the key person’s judgment will be less strict, and they are more likely to release substandard drugs. These results are consistent with previous studies.

### Personal commitments

Organizational commitments have a significant positive impact on deontological evaluation (H5 was accepted). Individuals with a higher level of organizational commitment have a higher level of recognition and a stronger sense of identity with the organization. The results show that key persons with higher organizational commitment would place a higher value on deontology. Key persons with high degrees of organizational commitment are more inclined to protect the organization through law-abiding behavior, leading to a more deontological approach to their evaluations.

Occupational commitments have a significant positive impact on deontological evaluations and a significant negative impact on teleological evaluations (H7 and H8 were accepted). Key persons with higher occupational commitment have a greater sense of identity with, and participation in their profession and give less consideration to the interests of other parties in making more rigorous ethical judgments. Key persons with high occupational commitment will identify more with their own occupations and pay more attention to their responsibilities. Therefore, they will closely adhere to the requirements of pharmaceutical laws and regulations.

### Comparison between major and minor deviations

According to the results of H-V core model in section 6.1, scenarios 1 and 2 have the same path of decision making. But the degree of the reject intentions is different. Paired sample T-tests were performed on the results of the two scenarios, results are shown in Table 7. The deontological evaluation (DE), teleological evaluation (TE), ethical judgment (EJ), and the reject intention (IT) of the two scenarios are significantly different. The teleological evaluation score for scenario 1 is significantly lower than for scenario 2. The other scores for scenario 1 are higher than for scenario 2. These results show that Scenarios 1 and 2 are different. When a key person faces serious deviations, they will more likely to use a deontological evaluation and have a higher intention towards drug rejection. However, when a key person faces minor deviations, they relax deontological evaluation while increase teleological evaluation. Key persons’ reject intentions appear to be lower when levels of deviation are low.

**Table 7:** Mean value comparison of Scenarios 1 and 2.

	Average of Scenario 1	Average of Scenario 2	Pairwise difference			
			Mean difference	Standard deviation	Standard error of mean	Sig. (two-tailed)
Deontological evaluation	6.211601	5.698529	.5130719	1.1653608	.0815916	.000
Teleological evaluation	2.433824	2.897059	−.4632353	1.2759122	.0893317	.000
Ethical judgment	5.799020	5.439216	.3598039	1.2784504	.0895094	.000
Reject intention	6.114379	5.619281	.4950980	1.5576771	.1090592	.000

## Conclusion and suggestions

Substandard drugs problems are observed around the globe. Therefore, research on its prevention has strong practical significance. With limited external regulator resources, internal controls from the key person for drug manufacturing sites are critical to fundamentally solve the substandard drug problem. This study discovered the consideration process when key persons reject a substandard drug and the factors affecting their reject decision.

EDM is used widely in business. This study provides empirical evidence on EDM H-V model used for substandard drug rejection. The results explain how key persons intention is influenced via ethical concerns and show that the EDM H-V model is suitable for explaining a key person’s intention to reject substandard drugs. The model explains that reject intention is significantly impacted by judgement. Key persons approach their reject judgement from both deontological and teleological perspectives.

Notably, this study unveils several new findings worthy of discussion. One of the major strengths of this research is the comparison of all results over two different levels of deviation from regulations. Key persons’ reject intentions appear to be lower when levels of deviation are low. The teleological evaluation score for minor deviation is significantly higher than for major deviation. When the key person faced to minor deviation form regulations, they are more teleological. They consider more from the consequences which may result from the adoption of each possible alternative. So more substandard drugs with minor deviation from manufacturing regulation are released by key persons.

This study provides a new individual factor for EDM study on professions with dual commitment. In both major and minor deviation scenarios, organizational and occupational commitments both have a significant impact on deontological evaluation, with the latter negatively impacting teleological evaluation. In reality, there are many occupations with dual commitment, such as teachers, doctors, lawyers, etc. They play important roles in society. Therefore, the results of this study provide a new research direction for their ethical decision-making research.

The results show that strengthening both occupational commitment and organizational commitment have significant effects on rejecting substandard drugs. In general, organizational commitment is an important part of training from the organization which is routinely practiced. Whereas, it is more difficult to strengthen occupational commitment at the workplace. QP’s management in China is a filing system which is difference from the qualification system in Europe. The manufacturing company selects QP by itself and records in the national management department. QP is a full-time employee of a manufacturing company and reports to the company’s senior leaders. Usually a manufacturing company has only one to two QPs, so it is difficult to develop the professional commitment of QPs[66]. Fortunately, there are many ways for the governing body to strengthen occupational commitment. For example, developing a key person written code of ethics similar to the Nightingale Declaration for nurses. Such a declaration would make key persons more aware of their mission.

In summary, this study shows that both occupational and organizational commitments are pivotal ethical factors that significantly impact the decision to release or reject substandard drugs. Fewer substandard drugs would be in the market by strengthening these commitments through various approaches.

## Limitations

This study only covered the ethical evaluation, judgement, and intention stages of H-V model. The behavior stage is not covered. According to H-V model, the behavior impact evaluation through the actual consequences. The action control and actual consequences will be studied in the future.

This study only focused on two individual factors, there are other individual factors which possibly impact substandard drugs release. Organization factor can also be covered, for example, the ethical culture, leadership style, organizational performance, rewards, competitiveness, and so on[48]. In future substandard drugs studies, more individual factors and organizational factors can be considered.

## Appendix: Scenarios

Scenario 1 *①Product A is an oral drug (4 mg/tablet)*. *②Recently*, *the company has been in a poor economic situation and has just received its first order in the last few months*. *③Unfortunately*, *the mixing equipment currently in use was damaged seriously*. *So in order to deliver on time*, *the production staff used the large-capacity equipment whose manufacturing process is still in study to increase the raw materials for mixing*, *fabricating the batch manufacturing records*. *④The test results of the finished products met the specification*. *No further test was conducted*. *⑤The QP was clear of the real manufacturing process and also knew the production records were fabricated*. *⑥He considered the quality risk of the drugs and the requirement of the company*, *then signed the release of this batch of products*. *⑦The company delivered drugs to the market in time*.
  *Notes: ①-⑦ represents the items for content validity.
Scenario 2 *①A company has just replaced the original equipment by a new set of equipment with the same model*. *②Equipment qualification is still in progress*. *③The company received an urgent order*, *a batch of products produced with the new equipment needed to be released today*. *④The person in charge of the equipment reported to QP*: *the equipment qualification data had not been completed and the formal equipment qualification report would take several days to complete*. *⑤The test results of this batch met the specification*. *⑥ QP considered the possibility that there exist problems in the product quality then signed the release of this batch of products*. *⑦The company delivered drugs to the market in time*.
  *Notes: ①-⑦ represents the items for content validity.

## Supporting information

S1 Table Data PLOS One. (PDF)
