# Supplementary material for: Key person ethical decision-making and substandard drugs rejection intentions
Source: PLoS One. 2020 Mar 19;15(3):e0229412. doi: 10.1371/journal.pone.0229412 (PMC7081989; doi:10.1371/journal.pone.0229412)
Supplement: S1 Table — (PDF) [file pone.0229412.s001.pdf]

	Gender	Age	Education	Employment length	Organizational commitment	Occupational commitment	1Deontological evaluation	1Teleological evaluation	1Ethical judgment	1Reject intention	2Deontological evaluation	2Teleological evaluation	2Ethical judgment	2Reject intention
1	2	4	1	1	6.4	7.0	7.0	1.0	7.0	7.0	7.0	1.0	7.0	7.0
2	2	2	2	2	5.2	6.2	7.0	2.0	5.8	7.0	6.3	2.5	5.2	6.3
3	1	3	2	2	4.8	5.7	5.8	3.7	6.4	7.0	1.7	5.3	1.4	1.0
4	2	2	2	1	5.6	7.0	6.0	3.2	5.2	4.0	5.5	3.8	5.4	6.0
5	1	3	2	4	5.4	3.3	4.8	3.2	4.6	5.0	4.7	3.3	4.6	5.0
6	1	4	1	4	4.4	5.8	6.5	1.3	5.6	7.0	5.2	2.0	3.8	6.3
7	2	3	1	4	3.6	7.0	7.0	1.5	7.0	5.0	7.0	1.0	6.4	7.0
8	2	3	1	4	3.4	5.0	5.0	3.5	4.6	5.0	4.8	3.7	4.6	5.0
9	2	3	2	4	6	5.7	7.0	1.0	7.0	7.0	7.0	2.0	7.0	7.0
10	1	2	2	3	3.4	7.0	7.0	1.0	7.0	7.0	7.0	1.0	7.0	7.0
11	1	3	2	1	6.6	6.5	6.7	2.5	6.0	7.0	6.8	1.3	6.8	7.0
12	2	4	2	1	3.8	4.0	4.0	5.8	4.4	3.7	4.0	5.0	3.4	2.7
13	1	3	2	3	3.2	4.0	4.5	6.0	3.8	4.3	4.0	6.3	4.2	3.7
14	2	3	3	3	3.6	4.0	3.7	6.2	4.4	3.7	3.8	5.7	3.4	2.3
15	2	2	2	1	3.8	5.0	6.0	2.7	6.0	7.0	7.0	1.0	6.4	7.0
16	2	3	1	4	4.8	5.8	6.8	2.0	6.0	6.0	6.0	2.7	6.0	6.0
17	1	3	1	4	6.6	6.5	6.0	1.0	5.6	7.0	6.8	1.0	5.6	6.7
18	1	2	2	4	6.6	7.0	7.0	1.0	7.0	7.0	7.0	1.0	7.0	7.0
19	1	2	2	4	4.6	5.7	5.7	2.0	6.0	5.7	3.5	3.0	5.0	5.0
20	2	4	2	4	5.4	3.8	7.0	1.0	7.0	7.0	7.0	1.0	7.0	7.0
21	1	2	2	3	6.4	6.5	7.0	5.7	4.8	6.0	5.0	7.0	4.6	7.0
22	2	2	2	1	6.4	6.7	7.0	1.0	7.0	7.0	7.0	1.0	7.0	7.0
23	1	2	3	3	5.6	7.0	7.0	1.0	7.0	7.0	7.0	1.0	7.0	7.0
24	1	4	2	3	4.8	6.0	6.2	1.8	5.8	6.0	6.0	3.5	5.4	6.0
25	2	2	3	2	5.2	4.8	4.8	3.2	5.4	6.3	6.0	3.5	6.0	6.0
26	1	2	3	3	6.8	7.0	5.5	3.8	6.8	5.0	5.2	4.0	5.6	5.0
27	1	2	2	3	4.2	7.0	7.0	2.0	6.2	5.0	7.0	1.0	4.6	5.3
28	1	3	1	3	7	7.0	7.0	5.0	5.8	7.0	7.0	1.0	7.0	5.0
29	1	1	1	3	7	7.0	7.0	1.0	7.0	7.0	7.0	1.0	7.0	7.0
30	1	2	2	3	5.4	6.0	5.8	3.5	5.6	6.0	5.7	2.3	6.0	6.0
31	1	2	2	3	7	7.0	6.0	1.0	7.0	7.0	7.0	1.0	5.8	7.0
32	2	2	1	2	7	7.0	5.7	3.8	5.0	5.0	4.7	4.3	4.4	5.0
33	1	3	1	3	7	7.0	6.0	3.3	6.2	5.0	4.0	6.0	3.6	3.3
34	2	2	1	3	7	5.0	7.0	1.0	7.0	7.0	7.0	1.0	7.0	7.0
35	2	2	1	4	6.2	5.5	5.8	4.0	5.0	6.0	5.7	4.2	4.0	4.7
36	1	2	1	4	7	6.5	6.7	1.0	6.6	7.0	5.8	4.8	3.0	2.3
37	1	2	1	1	5.4	6.8	7.0	1.0	7.0	7.0	7.0	1.0	5.8	7.0
38	1	2	1	4	5.8	7.0	5.8	2.5	4.6	7.0	6.0	3.0	5.2	7.0
39	2	1	2	3	6.8	6.2	7.0	1.0	7.0	7.0	7.0	1.0	7.0	7.0
40	1	1	1	1	5.8	6.0	4.0	7.0	5.0	5.7	4.0	6.3	4.2	3.0
41	2	2	1	3	6.6	7.0	7.0	1.2	5.8	7.0	6.8	2.2	6.0	7.0
42	2	1	1	3	6.6	6.7	7.0	1.0	7.0	7.0	7.0	1.0	7.0	7.0
43	2	1	1	3	6	7.0	7.0	1.0	7.0	7.0	7.0	1.0	7.0	7.0
44	1	2	1	4	7	7.0	6.0	3.0	5.8	7.0	7.0	1.0	7.0	7.0
45	1	2	1	1	7	7.0	7.0	1.0	5.8	7.0	7.0	1.0	7.0	7.0
46	2	3	1	4	7	7.0	7.0	1.0	7.0	7.0	7.0	1.0	7.0	7.0
47	2	4	3	3	6.2	2.7	7.0	2.3	6.4	7.0	4.7	5.8	2.6	4.7
48	2	4	1	4	7	7.0	7.0	1.0	7.0	7.0	7.0	1.0	7.0	7.0

49	1	3	2	4	7	7.0	7.0	1.0	7.0	7.0	7.0	1.0	7.0	7.0
50	2	2	1	3	7	6.0	4.0	5.0	7.0	7.0	7.0	3.0	7.0	7.0
51	1	2	2	3	4.6	5.8	6.5	1.8	6.6	7.0	6.5	1.8	6.4	6.3
52	1	1	1	2	4.6	5.7	6.0	1.0	7.0	7.0	7.0	1.0	7.0	7.0
53	1	2	1	4	6.6	6.8	7.0	2.0	6.0	7.0	7.0	2.0	6.0	7.0
54	2	2	1	3	4.4	5.5	7.0	1.3	7.0	7.0	7.0	1.0	7.0	7.0
55	1	3	4	3	4.6	6.3	6.0	1.0	5.8	7.0	7.0	1.0	7.0	7.0
56	1	2	2	1	7	7.0	6.5	3.3	4.4	7.0	7.0	3.8	5.4	6.7
57	2	4	2	4	6.8	6.0	7.0	1.0	5.8	7.0	6.7	1.8	6.2	5.0
58	2	3	1	4	4.6	7.0	7.0	1.3	6.6	7.0	6.2	3.3	5.0	6.3
59	2	3	1	4	5	7.0	6.3	4.7	5.6	5.0	3.3	5.2	4.0	4.7
60	2	4	3	4	5.8	2.0	6.0	3.7	4.8	6.7	6.0	4.3	4.6	6.7
61	1	3	2	4	5	5.3	7.0	2.2	5.2	6.0	5.5	3.7	5.0	5.0
62	1	2	3	2	5.8	7.0	7.0	1.0	7.0	7.0	6.0	1.0	7.0	7.0
63	1	2	3	1	2.8	3.8	6.0	1.0	7.0	7.0	6.0	1.0	7.0	7.0
64	2	2	3	1	3.6	5.0	7.0	1.0	7.0	7.0	7.0	1.0	7.0	7.0
65	2	4	3	4	6.2	6.5	6.0	4.0	5.2	6.7	6.7	3.7	5.4	6.7
66	2	2	3	4	6.2	5.3	6.0	1.0	5.8	7.0	5.2	1.0	6.6	7.0
67	1	1	2	1	6.6	6.0	5.7	2.0	6.0	6.0	4.7	4.8	3.0	3.0
68	2	3	1	4	5.8	5.7	7.0	1.0	7.0	7.0	6.0	1.0	7.0	7.0
69	2	2	2	3	6.4	7.0	6.8	5.0	4.4	4.7	6.5	3.3	5.2	6.0
70	1	4	3	4	6.6	3.8	6.5	2.0	6.4	7.0	7.0	1.0	6.4	7.0
71	2	2	2	2	5	4.7	6.8	3.0	6.4	6.3	6.2	3.7	6.0	7.0
72	2	2	2	2	4.8	5.3	5.0	1.0	5.6	7.0	5.0	1.0	4.6	7.0
73	2	3	3	4	7	7.0	7.0	1.2	6.2	7.0	7.0	1.8	5.0	7.0
74	1	3	1	1	6	6.3	6.2	3.5	5.2	6.0	6.0	4.0	5.2	6.0
75	2	3	1	4	6.8	7.0	6.0	1.7	4.8	6.7	7.0	1.7	4.8	5.7
76	1	3	2	1	6.6	4.8	4.2	3.7	3.4	4.3	4.5	4.0	3.8	4.0
77	2	3	2	4	6.8	5.3	6.0	2.0	6.0	6.0	6.0	2.0	6.0	6.0
78	2	3	3	4	6.6	4.0	6.2	3.7	3.8	4.0	2.5	6.0	1.8	2.0
79	2	2	1	3	6.2	5.3	5.7	2.8	5.6	7.0	4.2	2.2	6.0	6.7
80	1	3	2	4	5.8	2.0	7.0	2.0	5.8	7.0	5.0	6.0	2.2	1.0
81	1	3	2	4	6	4.8	6.8	1.2	6.4	6.3	6.2	2.0	6.0	6.7
82	1	2	2	3	4.4	6.2	5.0	6.2	3.6	3.0	5.7	5.7	3.6	2.0
83	2	4	2	4	5.2	1.8	2.0	5.8	2.0	2.0	2.0	6.0	2.0	2.0
84	2	2	1	4	4	4.8	7.0	1.2	7.0	7.0	7.0	1.0	7.0	7.0
85	2	1	1	1	6.2	5.5	5.3	4.2	6.6	6.3	6.7	4.0	7.0	7.0
86	2	2	2	3	5.6	5.3	5.2	3.5	4.6	5.0	5.5	3.2	5.0	5.0
87	2	2	1	4	7	7.0	7.0	1.0	7.0	7.0	6.0	1.0	7.0	7.0
88	1	3	1	4	7	7.0	7.0	2.0	7.0	7.0	7.0	1.0	7.0	7.0
89	2	1	1	2	5.4	6.0	7.0	1.0	7.0	7.0	7.0	1.0	7.0	7.0
90	1	3	1	4	7	7.0	7.0	1.0	7.0	7.0	7.0	1.0	7.0	7.0
91	2	2	1	4	6.2	7.0	5.8	5.3	5.4	6.3	5.0	3.8	6.2	6.0
92	2	2	1	4	3.4	6.0	7.0	2.3	4.6	5.0	7.0	3.8	7.0	7.0
93	2	2	2	4	5.8	6.8	5.5	4.3	5.8	6.0	4.7	4.0	5.2	6.0
94	1	3	1	4	5.8	7.0	6.0	1.0	7.0	7.0	6.0	1.0	7.0	7.0
95	1	1	1	1	6.6	6.3	7.0	3.3	6.4	7.0	7.0	3.0	7.0	7.0
96	2	3	1	4	6.6	5.3	6.8	1.0	5.8	7.0	7.0	1.8	6.2	7.0
97	1	1	2	3	6.2	6.0	7.0	1.0	7.0	7.0	7.0	1.0	7.0	7.0
98	2	3	2	4	4.8	6.0	7.0	4.7	4.6	6.3	6.8	6.0	4.6	6.7
99	2	2	2	4	6	5.7	7.0	1.7	6.0	6.0	6.0	2.0	6.0	6.0
100	1	2	2	2	6.6	5.0	6.2	3.2	5.4	7.0	6.0	5.0	5.4	6.0
101	1	3	1	2	4.2	5.0	4.8	5.3	3.8	3.0	3.7	5.2	3.8	3.7
102	1	3	2	4	5.8	7.0	7.0	3.0	7.0	7.0	7.0	2.5	7.0	7.0
103	2	2	2	3	4.6	6.0	7.0	1.0	5.8	7.0	6.0	1.0	7.0	7.0
104	2	2	2	1	6.4	5.7	7.0	1.7	6.4	7.0	6.5	2.2	5.2	7.0

105	1	2	2	4	6	6.0	7.0	1.0	6.6	6.3	6.3	1.0	6.0	7.0
106	1	3	2	4	7	7.0	7.0	1.0	7.0	7.0	6.0	1.0	5.8	7.0
107	1	2	2	2	6	6.5	6.0	1.8	7.0	7.0	7.0	1.5	5.8	7.0
108	2	2	1	3	3.8	6.7	7.0	1.3	5.6	7.0	5.3	2.5	6.6	3.0
109	2	2	2	4	6.6	6.5	5.8	2.0	6.0	6.0	5.3	3.3	5.2	5.7
110	1	3	2	2	5.6	3.7	6.8	1.8	4.6	7.0	5.2	2.8	4.6	6.7
111	2	2	2	4	6.6	3.8	6.8	3.2	5.6	6.0	7.0	4.0	7.0	7.0
112	2	4	2	4	7	7.0	7.0	2.0	7.0	7.0	7.0	1.8	7.0	7.0
113	1	2	3	2	6.8	6.0	7.0	1.0	7.0	5.0	3.2	2.7	3.2	3.7
114	2	3	1	4	7	5.2	6.8	3.7	4.6	7.0	6.0	3.8	6.0	3.0
115	1	3	2	4	6.6	6.3	6.5	2.3	6.2	7.0	3.5	5.5	2.0	2.0
116	2	3	2	2	5	3.8	4.3	3.3	3.6	4.0	2.0	4.0	2.8	3.0
117	1	2	2	4	6.4	5.8	7.0	1.8	7.0	6.7	7.0	1.0	7.0	7.0
118	1	2	2	3	5.8	7.0	5.5	3.3	6.0	7.0	7.0	4.2	6.2	7.0
119	2	3	2	4	7	5.5	6.8	1.3	7.0	7.0	5.7	5.3	6.2	6.7
120	1	2	2	4	5.6	6.3	6.5	1.8	6.2	6.0	4.0	4.0	3.4	3.0
121	2	2	2	4	5.8	5.2	5.3	3.2	5.6	6.3	5.7	4.3	5.2	6.0
122	1	2	2	4	7	5.7	6.7	1.5	5.8	7.0	4.8	3.3	4.4	3.3
123	1	3	2	4	7	6.2	6.0	1.0	7.0	7.0	7.0	1.0	7.0	7.0
124	1	3	2	4	7	7.0	5.7	2.0	5.8	7.0	6.0	1.0	7.0	7.0
125	2	3	1	4	4.6	6.7	6.3	1.8	6.0	6.0	2.7	2.0	3.2	5.0
126	2	2	2	4	7	5.7	7.0	1.0	7.0	7.0	7.0	1.0	5.8	7.0
127	1	2	2	4	6	4.7	4.7	1.7	5.4	6.0	4.3	2.2	6.0	6.0
128	1	2	2	4	6	6.0	6.2	2.2	6.8	7.0	6.7	1.0	7.0	7.0
129	2	2	2	4	6.6	5.0	6.5	2.2	6.4	6.0	6.2	2.5	5.2	6.0
130	2	2	2	2	7	7.0	7.0	3.3	4.4	5.3	7.0	3.5	7.0	3.3
131	1	4	1	4	6	4.8	3.8	6.7	3.4	3.0	4.0	7.0	3.4	3.0
132	1	1	2	2	7	7.0	7.0	1.0	5.8	7.0	6.0	1.8	4.8	5.0
133	1	2	2	2	5.4	6.0	6.0	2.5	6.0	6.0	6.0	2.3	6.0	6.0
134	1	4	2	1	5	5.0	4.7	4.3	4.6	4.0	3.2	6.0	2.4	2.3
135	1	2	2	3	5.2	5.5	5.8	5.2	5.2	4.3	5.5	5.0	5.2	4.0
136	2	4	2	2	6.4	4.3	3.3	5.3	2.4	1.7	2.8	4.7	3.0	2.0
137	1	3	2	1	5.2	6.2	5.5	1.8	5.8	6.7	6.3	2.0	4.2	7.0
138	1	4	2	1	4	5.2	5.5	2.3	5.0	4.3	5.5	3.2	5.4	5.0
139	1	2	2	2	6.4	4.2	6.7	1.0	7.0	7.0	6.7	1.0	7.0	7.0
140	1	1	1	1	7	5.8	7.0	3.5	5.6	7.0	3.7	6.0	3.8	6.7
141	1	2	2	1	6.2	6.8	7.0	1.0	5.6	7.0	7.0	1.0	5.8	7.0
142	1	2	2	3	6.8	6.8	7.0	1.0	7.0	7.0	7.0	1.0	7.0	7.0
143	1	3	2	3	6	6.0	7.0	1.2	4.8	7.0	6.0	1.0	7.0	7.0
144	1	3	2	4	7	6.0	5.0	1.0	5.8	7.0	5.0	1.0	5.8	7.0
145	2	2	2	1	6	7.0	6.0	4.0	5.8	2.7	5.3	4.7	5.2	3.0
146	1	3	2	4	5	3.3	4.3	3.2	4.6	5.0	5.0	3.2	4.6	5.0
147	1	2	2	1	7	6.5	6.8	2.0	5.6	7.0	5.7	2.7	5.2	6.0
148	1	3	2	1	5	7.0	7.0	2.0	5.8	7.0	4.2	6.0	5.2	3.7
149	1	1	2	3	4	5.3	6.2	2.7	5.0	2.7	5.0	4.3	3.4	5.0
150	1	3	2	4	6.2	7.0	6.7	1.2	5.8	7.0	7.0	1.7	5.4	7.0
151	2	2	2	4	6.8	5.7	6.0	5.5	4.4	1.0	5.0	7.0	4.6	1.0
152	1	4	2	4	4.8	2.8	6.7	1.2	7.0	7.0	7.0	1.0	7.0	7.0
153	1	3	2	1	6.6	5.5	7.0	1.8	6.4	7.0	6.5	3.5	6.2	6.0
154	2	4	2	4	3.2	3.7	4.8	3.8	5.0	6.0	5.2	3.5	4.8	5.0
155	2	4	2	4	6.8	6.7	6.8	2.0	5.0	7.0	5.2	2.5	6.4	7.0
156	1	4	2	3	3.8	7.0	5.5	2.0	6.0	3.0	3.8	6.8	1.8	1.0
157	1	2	2	2	4.6	5.8	7.0	1.0	7.0	7.0	1.5	6.0	2.0	1.7
158	1	2	2	2	4.6	4.2	7.0	1.0	7.0	7.0	3.7	5.3	5.2	1.0
159	2	3	1	2	6.8	5.0	6.8	4.5	5.8	6.7	6.0	5.0	6.2	7.0
160	2	3	2	4	5.4	4.2	5.2	3.3	5.2	6.0	2.8	6.3	2.0	2.0

161	2	3	2	4	7	5.8	7.0	2.8	5.8	7.0	6.3	2.2	5.6	7.0
162	2	3	2	4	5	5.2	6.2	1.2	5.4	7.0	5.2	2.0	5.8	6.0
163	1	2	2	1	6	6.7	7.0	1.2	2.8	6.7	5.0	1.7	5.8	3.7
164	2	2	2	1	4.8	4.5	5.5	1.8	5.8	6.7	4.8	2.3	4.0	6.0
165	1	3	1	1	5.4	6.2	5.3	3.2	6.0	7.0	4.8	3.7	6.6	7.0
166	1	2	2	2	5	4.0	5.5	2.5	5.0	6.0	5.8	3.2	4.6	5.7
167	1	3	1	4	6.2	4.2	5.8	2.3	4.4	7.0	1.7	6.5	1.8	1.3
168	2	3	2	1	4.4	4.8	4.8	5.3	3.4	2.0	5.0	4.2	5.8	6.7
169	1	3	2	1	5.6	6.2	6.8	1.0	6.8	7.0	2.3	4.3	3.8	2.3
170	2	2	1	4	6	4.8	6.2	3.2	5.8	6.0	6.0	3.7	5.4	5.3
171	1	3	2	2	5.2	6.8	6.2	1.0	7.0	7.0	7.0	1.0	7.0	7.0
172	2	4	1	1	7	7.0	7.0	1.0	7.0	7.0	7.0	1.0	7.0	7.0
173	1	2	2	3	3.8	3.7	4.8	2.3	4.2	2.7	4.0	3.8	4.6	5.7
174	2	1	2	1	4.6	5.3	7.0	1.0	7.0	7.0	7.0	1.0	5.8	7.0
175	1	3	2	3	5.2	6.8	7.0	1.8	6.8	7.0	6.8	1.3	6.6	7.0
176	1	2	2	3	4	5.5	6.2	3.2	5.4	3.7	6.2	3.7	5.2	4.0
177	1	2	2	2	4.8	6.0	6.0	2.0	5.4	6.0	5.7	2.0	5.4	6.0
178	2	3	2	4	4.4	3.7	6.8	4.2	6.0	4.3	5.7	2.7	5.2	6.7
179	2	3	2	4	7	4.7	6.3	2.8	5.0	6.3	5.0	3.0	4.0	7.0
180	2	3	2	4	4.2	4.0	4.8	4.5	5.2	5.3	5.0	5.8	3.2	3.7
181	2	3	2	4	5.8	3.8	6.3	3.7	6.2	7.0	5.2	5.0	5.2	6.0
182	1	3	1	1	3.6	3.2	6.7	4.7	5.2	2.0	7.0	6.0	4.6	3.3
183	1	3	2	4	3.4	3.8	4.0	4.8	3.4	3.7	3.5	5.0	3.6	4.0
184	2	2	3	4	4.6	6.3	6.2	2.0	6.0	7.0	6.2	2.0	5.0	7.0
185	1	1	2	1	5.2	2.0	6.0	3.7	4.8	2.0	7.0	1.0	7.0	7.0
186	2	2	3	1	5	5.7	5.8	2.2	6.0	6.0	3.3	3.8	4.0	4.0
187	1	1	3	1	5.4	5.7	6.0	3.7	5.2	6.0	6.0	3.3	5.4	6.0
188	1	1	2	1	2.6	4.3	6.0	1.0	6.0	7.0	5.0	1.0	5.8	4.0
189	1	2	3	3	5.8	7.0	7.0	1.0	7.0	7.0	7.0	1.0	7.0	7.0
190	1	1	3	1	5.4	6.0	7.0	2.8	6.8	7.0	5.2	4.8	4.8	4.0
191	1	1	3	1	4.8	5.8	7.0	1.0	4.6	7.0	6.0	1.0	7.0	7.0
192	1	2	3	1	4.4	4.3	5.7	3.7	5.4	6.3	5.5	4.3	5.8	6.0
193	1	2	3	1	3.8	4.2	4.2	4.8	3.4	4.0	3.7	4.8	3.4	3.0
194	2	1	3	1	4.8	5.2	6.8	1.2	5.8	7.0	6.0	1.0	7.0	5.0
195	1	2	2	2	6	6.7	5.8	2.3	4.6	5.7	6.2	2.0	4.8	6.0
196	2	1	2	1	6.8	6.8	7.0	1.0	7.0	7.0	7.0	2.3	6.6	6.3
197	2	1	2	1	7	7.0	7.0	2.0	7.0	7.0	7.0	1.0	7.0	7.0
198	1	2	2	1	4	4.0	5.2	3.7	5.0	4.0	5.0	3.0	4.0	4.0
199	1	4	2	2	6	5.3	5.5	2.0	6.0	6.0	6.0	2.0	5.2	6.0
200	2	4	1	4	4.4	3.0	6.7	2.2	5.4	7.0	4.0	6.0	2.8	2.3
201	1	4	1	4	7	6.8	7.0	2.0	6.4	6.7	7.0	2.5	7.0	7.0
202	1	2	2	1	5.4	6.2	6.2	2.7	5.4	4.0	5.5	3.8	5.4	4.0
203	1	4	1	3	7	7.0	5.0	1.0	5.8	7.0	6.0	1.0	7.0	7.0
204	2	2	2	3	3.4	4.3	5.7	3.2	5.2	3.7	3.8	4.3	3.6	2.7
